# High-energy brain dynamics during anesthesia-induced unconsciousness

**DOI:** 10.1162/NETN_a_00023

**Published:** 2017-12-01

**Authors:** James R. Riehl, Ben J. Palanca, ShiNung Ching

**Affiliations:** Department of Electrical and Systems Engineering, Washington University in St. Louis, St. Louis, MO, USA; Department of Biomedical Engineering, Washington University in St. Louis, St. Louis, MO, USA; Department of Anesthesiology, Washington University School of Medicine, St. Louis, MO, USA; Division of Biology and Biomedical Sciences, Washington University School of Medicine, St. Louis, MO, USA

**Keywords:** Network dynamics, Functional connectivity, Free energy, Resting-state networks, General anesthesia, Consciousness

## Abstract

Characterizing anesthesia-induced alterations to brain network dynamics provides a powerful framework to understand the neural mechanisms of unconsciousness. To this end, increased attention has been directed at how anesthetic drugs alter the functional connectivity between brain regions as defined through neuroimaging. However, the effects of anesthesia on temporal dynamics at functional network scales is less well understood. Here, we examine such dynamics in view of the free-energy principle, which postulates that brain dynamics tend to promote lower energy (more organized) states. We specifically engaged the hypothesis that such low-energy states play an important role in maintaining conscious awareness. To investigate this hypothesis, we analyzed resting-state BOLD fMRI data from human volunteers during wakefulness and under sevoflurane general anesthesia. Our approach, which extends an idea previously used in the characterization of neuron-scale populations, involves thresholding the BOLD time series and using a normalized Hamiltonian energy function derived from the Ising model. Our major finding is that the brain spends significantly more time in lower energy states during eyes-closed wakefulness than during general anesthesia. This effect is especially pronounced in networks thought to be critical for maintaining awareness, suggesting a crucial cognitive role for both the structure and the dynamical landscape of these networks.

## Author Summary

We show that activity in the human brain, as captured by functional magnetic resonance imaging (fMRI), is more organized during wakefulness than during general anesthesia. This increased organization corresponds to a decrease in a statistical-physics-inspired energy measure among brain regions of shared functional specialization (resting-state networks) that have putative roles in conscious awareness and attention. Characterizing the energy distributions in this way reveals significant changes in the dynamics of brain activity in different states of consciousness, insights that are not observable in the average functional connectivity data alone. Our results indicate that the ability of brain networks to sustain stable representations, via their dynamics, may be crucial for consciousness and cognition.

## INTRODUCTION

While general anesthesia has a seemingly unambiguous behavioral endpoint— unconsciousness—the neural mechanisms by which this state is achieved are diverse and highly enigmatic (Alkire & Miller, 2005; Brown, Lydic, & Schiff, 2010; Brown, Purdon, & VanDort, 2011; Mashour & Alkire, 2013). Thus, motivated by the premise that consciousness and cognition rely on a measure of coordination across brain regions and temporal scales, substantial effort has been directed at examining the effects of anesthetics on networks in the brain (Hudetz, 2012; Noirhomme et al., 2010; Peltier et al., 2005). In this regard, assessing the ability of brain networks to support and transition between a diversity of stable states is of paramount interest; such dynamics are thought to be key mediators of robust information processing and may be important in understanding fluctuating states in sleep and pathologic disorders of consciousness (Deco & Jirsa, 2012; Golos, Jirsa, & Daucé, 2015; Kinouchi & Copelli, 2006). The primary goal of this paper is to examine the effect of anesthesia on brain network dynamics through a statistical physics notion of energy and, specifically, to assess whether the state of unconsciousness is associated with an altered energy distribution relative to that of wakefulness.

More generally, collective organization measures such as energy and entropy have proven valuable in the analysis of brain activity patterns and in relating of such patterns to cognitive function. Indeed, the two most prevalent principles regarding maximum entropy and minimum energy are closely related in the context of brain dynamics. The maximum entropy principle states that a set of observations should be described by the distribution having the highest entropy (Jaynes, 1957). Put simply, a model for observed neural activity should assume as little as possible beyond what is represented in the data. It turns out that the maximum entropy model for a binary state system consisting of only pairwise interactions is known as the Ising model (Schneidman, Berry, Segev, & Bialek, 2006). This model was originally proposed to address phase transitions in networks of quantum spin states, but has since attracted considerable attention in computational neuroscience (Cocco, Leibler, & Monasson, 2009; Roudi, Tyrcha, & Hertz, 2009). Note that in this context, entropy is largely a static concept insofar as it describes a distribution of activation states, and it provides little information about the dynamics of how those states evolve over time. On the other hand, the free-energy principle, which postulates that a self-organizing system tends to minimize free or excess energy (K. Friston, 2010), is primarily a statement about dynamics. The Ising Hamiltonian, which can be thought of as a measure of free energy in a binary state system, is a dynamic quantity expressing the degree to which neighboring nodes or regions are in alignment over time (Ising, 1925). For a pair of brain regions in which activity is positively correlated, alignment means that these regions are either both active or both inactive at a particular time, while for anticorrelated regions, alignment means that they occupy opposite activation states. As the Ising energy of a network decreases, it moves closer to a state of equilibrium. When invoking the free-energy principle, we do not claim that the brain ever reaches or even closely approaches such equilibria. Rather, the overall premise of this paper is that by observing the distribution of these energies over time, it may be possible to broadly characterize different modes of functionality in the brain. To investigate this issue, we will examine how the energy landscapes of brain networks, defined from functional magnetic resonance imaging (fMRI) data, vary between subjects who are awake and under general anesthesia.

Variations on the Ising model have been used previously to model emergent properties of brain networks at the neuronal level. In this setting, individual neurons can be modeled as being either active or inactive, thus enabling characterization of network statistical properties within the Ising formalism (Cocco et al., 2009; Hopfield, 1982; Roxin, Riecke, & Solla, 2004; Schneidman et al., 2006). However, there have been considerably fewer investigations of Ising energy at the broader spatial level of connectivity between brain regions, which is our interest herein.

Perhaps the most common analysis of brain network at broad spatial scales involves computing the functional connectivity, wherein the relationship between brain regions is characterized in terms of the covariance of their time-varying signals (K. J. Friston, 1994; Jiang, He,Zang, & Weng, 2004; Mayer, Mannell, Ling, Gasparovic, & Yeo, 2011; Wang et al., 2007). Related measures, such as the Pearson correlation coefficient, can be projected as edge weights in a network, taking either positive or negative values depending on whether the corresponding regions are correlated or anticorrelated. These relationships have been used to define resting-state networks (RSNs), parcels of brain regions whose patterns of correlated signal reflect shared functional specialization during task performance (Beckmann, DeLuca, Devlin, & Smith, 2005; Cordes et al., 2000; Smith et al., 2009). By returning to the time series data after computing the functional connectivity matrices, we take this line of analysis a step further and characterize the brain’s energy distribution over time.

Energy-based analysis can further illuminate properties of the underlying brain network dynamics, even at the coarser spatial resolutions of fMRI. For example, energy distributions on networks built from resting-state fMRI data have been modeled using a stochastic gradient descent algorithm to find local energy minima (regarded as equilibria of the underlying network dynamics) and estimate their corresponding basins of attraction (Gu et al., 2016). Indeed, this energy characterization revealed positive correlations between observed and predicted rates of regional activation. Our objective is to examine how alterations in the energy landscape may relate to a systematic alteration in cognitive state (namely, a state of general anesthesia). In this regard, it has been predicted from simulations that the awake resting (i.e., unanesthetized) state corresponds to network states that are closer to equilibrium (Hudetz, Humphries, & Binder, 2014).

The main contribution of our work is that, for the first time, we provide empirical evidence that wakeful consciousness correlates to the brain spending more time in lower energy states than under general anesthesia. To do so, we introduce a correlation-normalized Hamiltonian, in order to assay the energy landscapes in a manner that is invariant to the overall strength of functional connectivity. We observe the effect across a majority of RSNs, and notably in the default mode and somatomotor networks. In contrast, the energy landscapes of the vision and language RSNs were mostly robust to general anesthesia.

## RESULTS

### Global Reduction in Functional Connectivity During General Anesthesia

**Figure 1.**
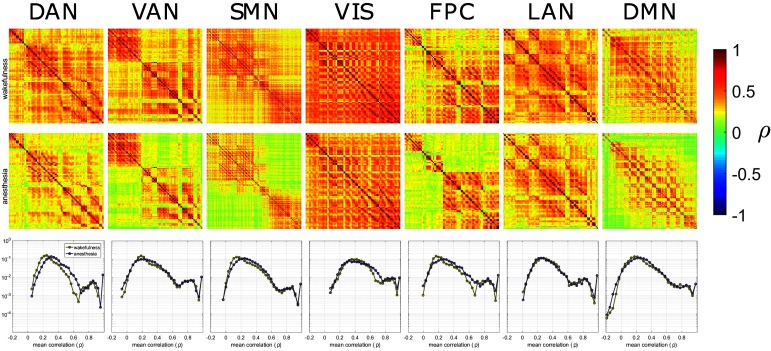
Correlation matrices (matrices of Pearson correlation coefficient *ρ*) averaged over all subjects in states of wakefulness (top) and general anesthesia (middle) for each RSN. Note that the number of regions varies across RSNs. Although the majority of correlations are positive, there are indeed scattered weak anticorrelations between regions, as shown in the histograms on the bottom row.

**Figure 2.**
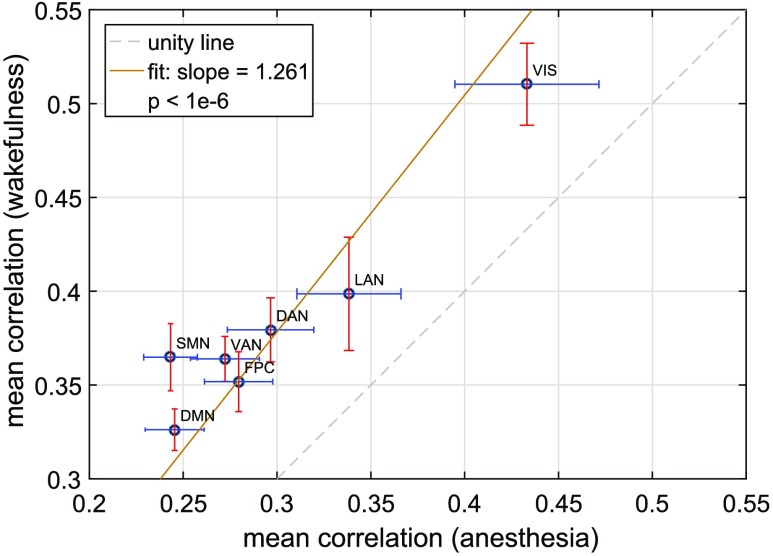
Linear regression through the origin reveals that the mean correlation coefficients during wakefulness are higher than those during general anesthesia in relatively consistent proportions across RSNs.

Our analyses focus on within-RSN functional connectivity and energy dynamics. Figure 1 shows the functional connectivity matrices for each of the seven defined RSNs, averaged over all subjects (see Materials and Methods). The top row corresponds to the awake resting state, while the second row corresponds to general anesthesia. While most correlations are positive, there exist scattered weak anticorrelations between regions, which can be seen in the histograms on the bottom row of Figure 1. We may surmise from these matrices that the overall connectivity patterns are similar between the two conditions. If we perform a linear regression relating the mean correlation coefficients during wakefulness and under general anesthesia, shown in Figure 2, we see that the mean functional connectivity during wakefulness is approximately 1.26 times stronger than that during general anesthesia. Although there is some small variation across the RSNs, particularly in the somatomotor network, the overall effect is relatively consistent across brain regions, a known phenomenon associated with general anesthesia (Boveroux et al., 2010; Palanca et al., 2015). In what follows, by characterizing the energies of the BOLD time series data in the context of these functional connectivity networks, we provide additional insight into the how the dynamics of the brain are affected by general anesthesia and how these effects vary across RSNs.

### An Anesthesia-Induced Shift to High-Energy Dynamics

**Figure 3.**
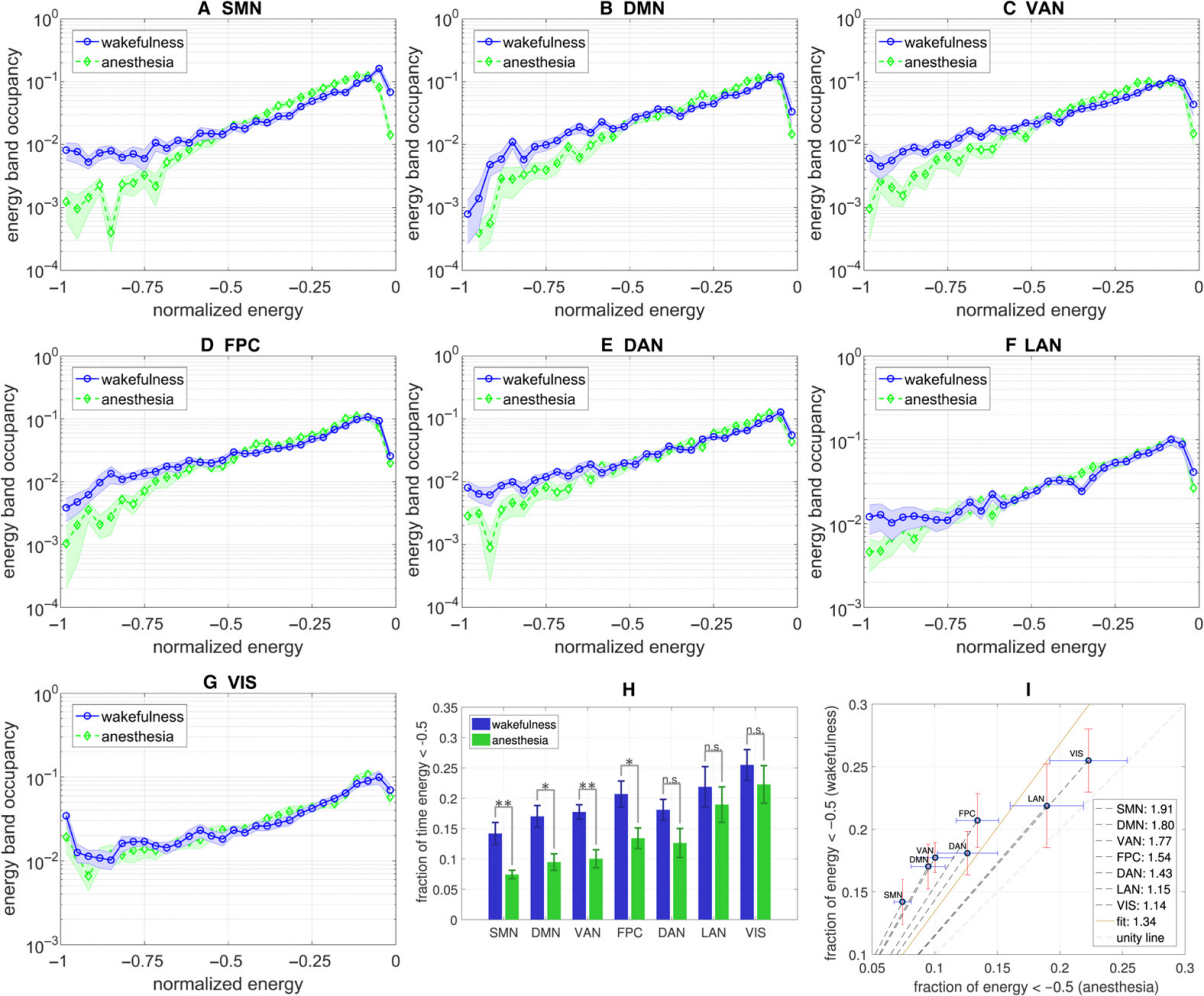
The difference in energy distribution during wakefulness and general anesthesia for each RSN (A–G). Summarized in the bar plot H, wakefulness correlates to the brain spending significantly more time in lower energies than during general anesthesia. In paired-sample *t* tests, * indicates that *p* < 0.1 while ** indicates that *p* < 0.01. Note that by convention the Ising Hamiltonian becomes more negative with decreasing energy. The strengths of these effects are quantified in the slopes of plot I, listed in the legend. The networks with the strongest effects are DMN and SMN, whereas the landscapes of LAN and VIS are more invariant to anesthesia.

Since sevoflurane anesthesia appears to globally weaken intracortical functional connectivity, we proceeded to compute the Ising energies according to a normalized Hamiltonian function (that is invariant to a uniform scaling in correlation; see Methods). Figure 3A–G shows the resulting energy distributions on a normalized abscissa for each of the seven different RSNs. The plots depict a trend in which the energy landscape under general anesthesia has less density in the lower range of normalized energies versus that of wakefulness (as is the convention, the Ising Hamiltonian becomes more negative with decreasing energy). Specifically, when computing the relative densities in the lesser (< 0.5) versus greater half (> 0.5) of the normalized energy distributions, as summarized in Figure 3H–I, we observe a robust effect wherein the human brain spends more time in lesser-half energy states during wakefulness than during anesthesia. This result holds across most RSNs, with the exception of the language, vision, and dorsal attention networks, in which the effect is not statistically significant, that is, the *p* values resulting from paired-sample *t* tests were greater that 0.1. The magnitude of the difference is largest in the somatomotor and default mode networks, indicating that the dynamics of these networks may be more susceptible to sevoflurane or generally may play an important role in maintaining conscious awareness (see Discussion).

Comparing the slopes in Figure 3I to those in Figure 2, we see that the relative change in functional connectivity is generally smaller than the change in energy distribution and is also not necessarily a good predictor of this change. Hence, the energy characterization indeed provides information that cannot be determined directly from the correlation matrices.

### Low-Energy States Are Relatively More Frequent Within RSNs Than at the Global Level

**Figure 4.**
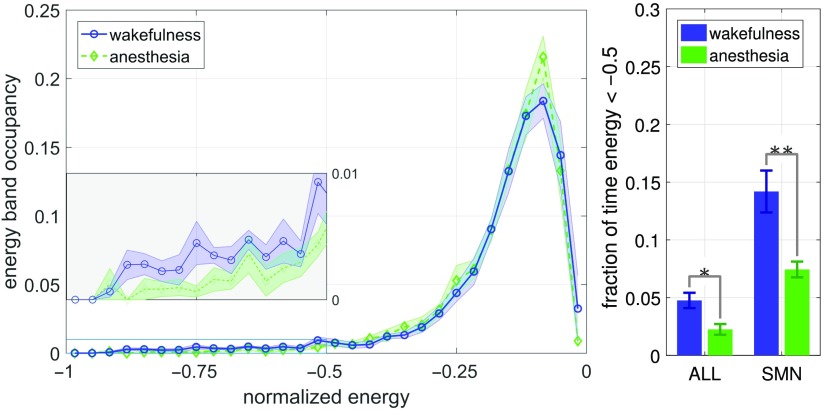
Energy distributions of the aggregated seven-RSN network during wakefulness and general anesthesia, averaged over all subjects. A linear scale is used here for the y-axis since zero values cause portions of the data to be undefined on the log scale. We see in the bar plot that the fraction of time spent in lesser-half energies is much smaller for the aggregate network than at the RSN level (SMN shown for comparison), although the ratio of these quantities between wakefulness and anesthesia is comparable across scales.

We also computed the energy landscapes of the aggregated network consisting of all seven identified RSNs averaged over the nine subjects under each condition. Figure 4 shows a histogram of the results comparing wakefulness to general anesthesia. We observe in the bar plot, comparing to SMN from Figure 3H, that the amount of time spent in lesser-half versus greater-half energy states is much larger at the RSN level than globally. However, the relative proportion of time spent in lesser-half energy states during wakefulness versus general anesthesia is consistent between both scales. This suggests that within-RSN brain activity is more likely to hold patterns closer to equilibrium when compared with global brain activity, but that general anesthesia correlates to similar relative energy changes at both scales.

## DISCUSSION

Our results represent the first demonstration that general anesthesia alters the energy landscape of neural dynamics. We specifically showed that key resting-state networks in humans exhibit a pronounced shift to higher energy dynamics during unconsciousness. There are diverse theories regarding the mechanisms of anesthesia-induced unconsciousness, though many coalesce around notions of hierarchy, synchrony, and integration within and between cortical and subcortical brain networks (Brown et al., 2011; Lewis et al., 2012; Mashour & Alkire, 2013; Tononi & Edelman, 1998). We have shown here that at broad spatial scales and at second-to-second timescales accessible through fMRI, anesthetic-induced unconsciousness is associated with patterns of cortical activity that are further from equilibrium. This suggests that the underlying dynamics are less capable of supporting stable configurations. Such dynamics are thought to be instrumental for efficient information processing and mentation (Hudetz et al., 2014; Hudson, Calderon, Pfaff, & Proekt, 2014; Kitzbichler, Smith, Christensen, & Bullmore, 2009), and thus the alterations to energy that we show here—reflective of a transition toward instability—are readily reconcilable with the state of unconsciousness.

### Energy, Entropy, and Complexity

We note that our analyses are based on the physics framework of energy/order rather than on the intensity of metabolic activity (i.e., physiological energy) as assayed, for instance, via positron emission tomography. During general anesthesia, reductions in cerebral metabolic rate and oxygen consumption are reproducible phenomena (Hirsch & Taylor, 2016).

Since there is significant literature on entropic properties of brain activity in the context of general anesthesia, especially over faster timescales (e.g., Liang et al., 2015), it is worth discussing how these results differ from an entropy-based analysis. One practical reason for using energies rather than entropies in this context is that the entropies can be numerically problematic to compute. An early step in computing the entropy is to fit the data to a probability distribution such as a multivariate Gaussian. In this case, given a covariance matrix Σ for the Gaussian fit, the resulting entropy is given by $\frac{1}{2}\ln(|2\pi e\Sigma |)$. However, since the empirical covariance matrices are poorly conditioned (≈ 10^18^), these determinants and corresponding entropies are generally very small and of questionable value. Computing the energies as we have done by means of transformation to a binary state system enables a tractable analysis. Furthermore, these measures are interpretable in terms of the free-energy principle for dynamic activity in the brain.

Nevertheless, our analysis here can be viewed as a type of complexity characterization since lower Ising energies may be interpreted as more organized (or, less “complex”) activity over time. From this perspective, one might infer from our results that sevoflurane anesthesia induces more randomness and thus more complexity of brain dynamics, which stands in contrast to previous findings and hypotheses that associate unconsciousness (including states of general anesthesia) with a decrease in complexity (Tononi, 2004; Tononi & Edelman, 1998). For example, the entropic brain hypothesis (Carhart-Harris et al., 2014) suggests that complexity constrains the level of consciousness, with higher complexity enabling more primitive conscious experience, while lower complexity is presumably associated with conditions such as anesthesia and deep sleep. It should be noted that some of these findings, such as Solovey et al. (2015), are based on electrophysiological recordings sampled at much higher rates than the second-to-second timescales we consider herein. However, there is also fMRI-based evidence of increased stability during unconsciousness (Tagliazucchi et al., 2016). Also in the fMRI domain, Hudetz, Liu, and Pillay (2015) and Hudetz, Liu, Pillay, Boly, and Tononi (2016) associated wakeful consciousness with a larger repertoire of brain states and thus higher entropy when compared with propofol anesthesia. What seems at first glance to be a conflict between these prior descriptions and our current results reveals upon closer inspection some subtle but important distinctions between the concepts of energy, entropy, complexity, and stability in the context of brain dynamics. These descriptors, while conceptually related, do have distinct technical meanings. It is important to emphasize that the Ising energy spectrum holistically describes the fraction of time spent in states that are (mis)aligned with the average functional connectivity. This concept of energy is thus not a direct measure of entropy or stability and should not be interpreted as a surrogate for these other notions. In fact, it is quite possible that sevoflurane anesthesia correlates to both an increase in average Ising energy and a contraction in the repertoire of reachable states. In this case, the activity may be “simpler” in the sense of diversity of patterns manifest, but more apt to display temporal fluctuations. This would be consistent with the simulation results of Hudetz et al. (2014), in which increased activity in wakeful consciousness leads to a greater number of local state changes, which tends to both decrease the energy of the system while also expanding the range of states that can be explored. Our findings may be an empirical substantiation of this phenomenon. Thus, our characterization of normalized energy ultimately provides a description of brain dynamics that is complementary to existing theories of unconsciousness.

### Resting-State Networks and General Anesthesia

Our results indicate that the largest marker of sevoflurane-induced unconsciousness in energy distributions lies in the somatomotor and default mode networks. The former is thought to be involved in motor and somatosensory processing, though it has rarely been implicated in mechanisms of anesthetic unconsciousness. This finding is not surprising, however, as our surrogate for loss of consciousness is lack of motor responses to noxious stimulation. Disruption of dynamic cortical activity in somatomotor regions is a plausible correlate or mechanism for unresponsiveness. The latter finding regarding the default mode network is supported by several previous findings that the DMN is closely linked to conscious awareness. For example, Horovitz et al. (2009) found that sleep-induced reduction of consciousness correlates to significant changes in functional connectivity between DMN components, particularly with respect to the frontal cortex (Horovitz et al., 2009). In addition, Greicius et al. (2008) reported that low levels of conscious sedation are commensurate with weaker correlations between the posterior cingulate cortex and other regions of the DMN (Greicius et al., 2008). In a neurological study on patients with various degrees of conscious impairment, Vanhaudenhuyse et al. (2010) found negative correlations of clinical consciousness to connectivity in multiple areas of the DMN (Vanhaudenhuyse et al., 2010).

Strong effects on the energy landscape were also observed in the ventral attention network and frontoparietal control network, both of which are thought to be involved in initiating and modifying transient changes in attention and information processing (Dosenbach et al., 2007), the disruption of which is consistent with the state of unconsciousness. Our prior functional connectivity analyses also highlighted disruptions in the VAN (Palanca et al., 2015).

As previously mentioned, many prior studies have characterized widespread weakening in functional connectivity within and between resting-state networks during general anesthesia (e.g., Boveroux et al., 2010; Liu et al., 2012), though none have characterized network dynamics in terms of energy landscapes. It is of note that in these prior works, “low-level” sensory networks such as the visual network appear most robust to the effects of anesthesia, similar to what we observe in the energy landscapes of these networks. Since our results suggest that sevoflurane-induced unconsciousness results in an even greater change to the energy distributions than to functional connectivty at the RSN level (as evidenced in the slopes of Figures 3I and 2), an energy-based analysis is potentially an even more sensitive marker of clinical unconsciousness than direct functional connectivity measures.

### Robustness to Connection Density

**Figure 5.**
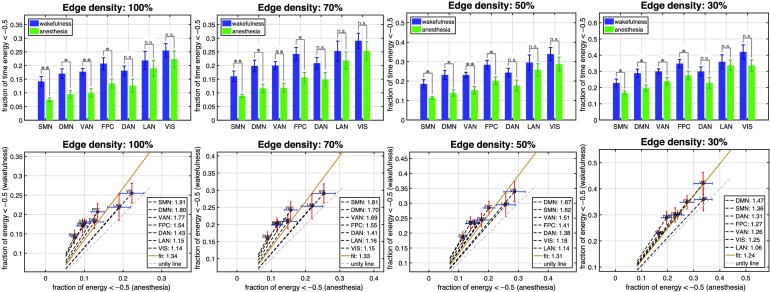
Thresholding the functional connectivity matrices to eliminate edges between weakly correlated regions does not qualitatively alter the results.

In our primary analysis, rather than thresholding the correlations between BOLD signals to eliminate weakly correlated edges in the functional connectivity networks, we assumed a completely connected network in which both strong and weak correlations may exist. Since our normalized energy measure weights the contribution of each edge by the correlation coefficient, weak correlations contribute relatively little to the total energy. However, there remains a risk that in aggregate, these correlations could induce a bias in the results. We therefore verified the robustness of our findings to this assumption by performing the analysis on a series of thresholded networks with varying edge densities, following methods similar to those used in Tagliazucchi et al. (2016). Figure 5 shows that the qualitative results are quite robust to the deletion of weakly correlated edges, as we continue to see significant differences between the energy distributions corresponding to wakefulness and anesthesia in the same RSNs as the complete network case. Although the magnitudes of the differences (indicated by the slopes in the figures on the bottom row) decrease as the networks become more sparse, this is to be expected since including only the most highly correlated edges will trivially reduce the frequency that connected pairs deviate from expected correlation across all RSNs in both wakeful and anesthetized states. For example, in the limit as the edge density decreases to include only a few almost perfectly correlated region pairs, which are indeed observed, the normalized energy distributions for both cases would converge to a peak around negative one and the corresponding difference in energies would therefore decrease to zero. The detailed energy distributions for each RSN in the thresholded cases are provided in the Supplementary Information (Riehl, Palanca, & Ching,, 2017).

The primary reason for using the normalized Ising Hamiltonian rather than the standard unnormalized one was to remove a potential source of bias due to alterations in functional connectivity between conditions of wakefulness and anesthesia. Since the correlations are lower across all RSNs during anesthesia, this would most likely have a direct effect on the energies, if not removed via normalization. Nevertheless, it may still be informative to see the difference in raw energies between the two conditions. Although difficult to compare with the normalized case, we observed qualitatively an even greater difference in energy distributions between the two conditions for most RSNs, with the exception of VIS and LAN (see the Supplementary Information for detailed figures; Riehl et al., 2017).

### Mechanisms of Energy Alteration

The mechanisms behind the dynamics driving the brain toward lower energy states remain uncertain. However, we do know some theoretical principles that govern any potential dynamics within this framework. First, the balance between correlated and anticorrelated brain regions plays a critical role in the resulting equilibria and convergence properties, and is closely related to excitatory and inhibitory connections. For example, networks consisting of entirely correlated or entirely anticorrelated activity tend to converge toward an equilibrium, but a mixture of both may remain in fluctuation even in the fully deterministic case (Ramazi, Riehl, & Cao, 2016). In the case of Hudetz et al. (2014), it appears that only positive correlation values were used, and the result was that widespread alignment was observed for the cases when switching was less random and more deterministic.

It is also worth mentioning that mutual excitation, mutual inhibition, and anticorrelation may all play significant roles in the functionality and thus energy landscapes of brain networks (Uddin, Clare Kelly, Biswal, Xavier Castellanos, & Milham, 2009). Although correlation (respectively anticorrelation) between regions does not necessarily imply excitatory (respectively inhibitory) activity in the underlying physiology, in order to include these effects, a model should be able to capture all three types of interactions listed above. While our framework indeed accounts for all three, some previous analyses compute energies based only on coactive regions, which may not be sufficient to capture the various types of interactions. For example, by choosing the activation state in {0, 1} rather than {−1, +1} while computing the energy using a Hamiltonian similar to Equation 3 (Gu et al., 2016), there is a risk of neglecting the significance of correlated brain regions being simultaneously inactive or inhibited.

### Limitations

While neuroimaging has been used extensively for characterizations of (stationary) functional connectivity, its use as a modality for examining brain dynamics remains limited because of the relatively coarse nature of the BOLD signal and low sampling rate. As a result, we must be careful in limiting the interpretation of our results to only the spatial scale of RSNs and dynamics over relatively long temporal epochs. The role of networks at finer spatiotemporal scales is beyond the explanatory power of our current data. We utilized global signal regression to minimize signal artifacts related to micromovements of the head, but this may have the additional effect of shifting the distribution of correlation coefficients toward negative values. We are also unable to evaluate the possibility that the apparent integrity of the language and visual RSNs reflect ongoing neural phenomena, such as dreaming.

Several caveats must be acknowledged regarding our use of sevoflurane, one of many drugs known to induce altered states of consciousness. As we have discussed in a previous study, precisely correlating loss of responsiveness with sevoflurane dose and concurrent BOLD fMRI is difficult in part because of movement artifacts incurred during sedation (sevoflurane %vol concentration 0.6–1.0%) (Palanca et al., 2015). As with other reports in this area, we are unable to control for the possibility that observations reflect anesthetic effects on the BOLD signal rather than loss of consciousness. At sedative doses, sevoflurane alters cerebral blood flow and BOLD signal amplitude in a heterogeneous manner across the cerebral cortex (Qiu, Ramani, Swetye, Rajeevan, & Constable, 2008). Similar studies have not been reported for sevoflurane concentrations used in this study. The possibility also remains that participants held at 1.2% sevoflurane may have retained elements of consciousness during portions of the fMRI data acquisition. While we operationalized the loss of consciousness endpoint by assessing the lack of withdrawal following noxious fingernail bed stimulation, we concede that multiple factors could lead to an inaccurate inference (Sanders, Tononi, Laureys, & Sleigh, 2012). Responsiveness may have been ablated in the context of an intact consciousness through alteration of pain threshold or sensory processing, reduced motivation to respond, or perturbation in attention. Recent investigations suggest that even at doses of inhalational anesthetics comparable to what is used for surgical anesthesia, patients (Sanders et al., 2017) and volunteers can still exhibit responses to verbal command (Pavone et al., 2017). Thus, while loss of responsiveness was ascertained prior to scanning, whether this state persisted is unknown and remains a topic of ongoing investigation. Finally, it remains to be seen how well our results generalize to other states induced by anesthetics with different molecular mechanisms of action.

## CONCLUSION

In summary, we have provided empirical evidence that at the macroscopic scale the human brain spends significantly more time in lower energy, more organized states during wakefulness than during general anesthesia. This finding is consistent with the free-energy principle and the notion of low-energy states being important for neural information processing. Moreover, standard functional connectivity measures are not sufficient to arrive at these conclusions, indicating that energy-based analysis may be a valuable tool for characterizing macroscale brain dynamics in other cognitive states.

## MATERIALS AND METHODS

Our results build on a prior investigation of the BOLD functional connectivity changes associated with sevoflurane general anesthesia (Palanca et al., 2015) and use the same dataset.

### Ethics Statement

All data were collected with approval from the Human Research Protection Office at the Washington University School of Medicine. Written informed consent was obtained from all participants.

### Participants and Data

Briefly, resting-state blood-oxygen-level dependent (BOLD) functional magnetic resonance imaging (fMRI) data were acquired from nine healthy subjects during quiet wakefulness and from nine subjects rendered unresponsive by the anesthetic drug sevoflurane (1.2 vol%). Six of the subjects were imaged in both wakeful and unconscious states, meaning that data used for this study were collected from a total of 12 subjects. To ensure that no bias resulted from the inclusion of unpaired data from six volunteers, we also performed the analysis on only the six subjects with paired data. These results, included in the Supplementary Information (Riehl et al., 2017), turned out very similar to our main results, in some cases resulting in even greater contrast between wakeful and anesthetized conditions, indicating that any bias incurred from this choice was negligible. The volunteers breathed spontaneously through a mask during anesthesia. Unconsciousness was assessed by unresponsiveness to noxious nail bed pressure. Imaging was commenced after at least 15 min of equilibration to the sevoflurane. A Siemens 3T Trio MRI scan was used to acquire echoplanar BOLD images (4 mm isotropic voxels, TR 2200 ms, TE 27 ms, FOV 256 mm, FA 90 degrees, 36 slices/volume, 200 volumes per run).

A total of 3,715 frames (136 min) were collected from awake subjects and 3,131 frames (114 min) from unconscious subjects. A complete exposition regarding the neuroimaging equipment and scan procedures can be found in Palanca et al. (2015).

Data preprocessing was handled exactly as in Palanca et al. (2015) and detailed also in Kafashan, Ching, and Palanca (2016). Cortical gray matter was parcellated into 6 mm^3^ regions. We selected 1,076 regions on the basis of a winner-take-all algorithm for exclusive membership (Hacker et al., 2013) in the following resting-state networks: dorsal attention (DAN, 94 regions), ventral attention (VAN, 118 regions), somatomotor (SMN, 313 regions), visual (VIS, 105 regions), frontoparietal (FPC, 86 regions), language (LAN, 100 regions), default mode (DMN, 260 regions). All BOLD signals underwent denoising for motion artifact, through regression of whole-brain global signal and censoring of corrupted frames.

### Functional Connectivity Analysis

To estimate functional connectivity between brain regions, we calculated Pearson correlations between each pair of regions, generating a (symmetric) connection weighting matrix *J*, in which each entry *J*_*ij*_ is the correlation coefficient between regions *i* and *j*. Rather than thresholding the matrix to preserve only the strongest correlations, we assume a completely connected network in which both strong and weak correlations may exist. To ensure that no significant bias resulted from this assumption, we verified the robustness of our findings to network density by performing identical analyses on thresholded networks with varying edge densities (see Discussion: Robustness to Connection Density).

### Ising Energy Calculation and Normalization

Let ${\tilde{x}}_{i}(t)$ denote the BOLD fMRI contrast at a given region *i* and time *t*, and let ${\overset{-}{x}}_{i}$ denote the mean of ${\tilde{x}}_{i}$ over the recorded time. We define a quantized activation state *x*_*i*_(*t*) ∈{+1,−1} as follows:

$$x_{i}(t)=sign({\tilde{x}}_{i}(t)-{\overset{-}{x}}_{i}).$$ (1)

As shown in Schneidman et al. (2006), the maximum entropy model for a two-state system dominated by pairwise interactions is the Ising model. The dynamic energy is computed according to the Hamiltonian for the Ising model, which is given by

$$\begin{array}{l} H(x(t))=-\underset{i,j}{\sum }J_{ij}x_{i}(t)x_{j}(t), \end{array}$$ (2)

where *J*_*ij*_ denoted the Pearson correlation coefficient between regions *i* and *j*. At each time *t*, this energy is minimized for a given region pair {*i*, *j*} when the sign of *x*_*i*_(*t*)*x*_*j*_(*t*) is the same as the sign of correlation coefficient *J*_*ij*_. Namely, when regions *i* and *j* have positive correlation, then energy is minimized when these regions are either both active or both inactive. Conversely, when regions *i* and *j* are anticorrelated, energy is minimized when these regions are in opposite activation states.

Since we are primarily interested in comparing energy levels across RSNs and under different states of arousal, we prefer an energy measure that is independent of network size and total functional connectivity level. Therefore, we define the following normalized energy:

$$\begin{array}{l} Ĥ(x(t))=-\frac{\sum_{i,j}J_{ij}x_{i}(t)x_{j}(t)}{\sum_{i,j}|J_{ij}|}. \end{array}$$ (3)

**Figure 6.**
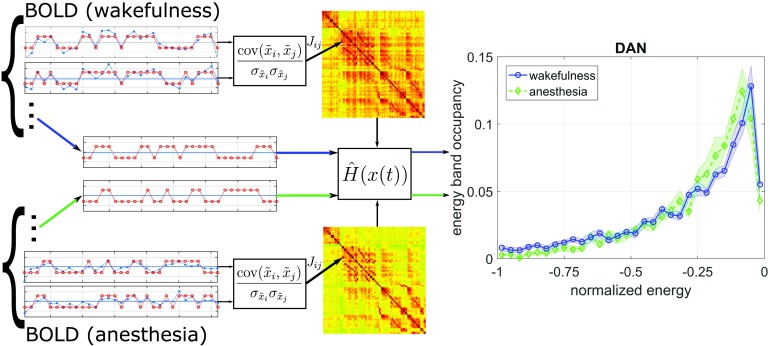
Diagram of the energy computation process. Average correlations between pairs of BOLD signal data populate the functional connectivity matrices. Binarized BOLD signals (red) are generated from BOLD time series (blue). The average correlations are in turn used to compute the energies of the binarized BOLD signal data, which are collected in histograms for each RSN.

Note that the resulting measure is invariant to arbitrary scaling of the *J* matrix. Consequently, the lower levels of functional connectivity observed during anesthesia when compared with wakefulness will not bias a comparison of energy distributions. For further discussion and a comparison of normalized versus unnormalized energy, see the Supplementary Information (Riehl et al., 2017). Figure 6 shows a schematic of the process described in this section, how we compute normalized energy distributions from BOLD signals.

## AUTHOR CONTRIBUTIONS

James R. Riehl: Conceptualization; Formal analysis; Investigation; Methodology; Validation; Visualization; Writing original draft; Writing review & editing. Ben J. Palanca: Data curation; Funding acquisition; Investigation; Validation; Writing review & editing. ShiNung Ching: Conceptualization; Funding acquisition; Investigation; Methodology; Project administration; Supervision; Validation; Visualization; Writing original draft; Writing review & editing.

## FUNDING INFORMATION

This work was partially supported by the U.S. Air Force Office of Scientific Research: 15RT0189 (SC); the National Science Foundation: ECCS-1509342 (SC), CMMI-1537015 (SC); the National Institutes of Health: 1R21NS096590-01A1 (SC), UL1 TR000448 (BP), KL2TR000450 (BP), R21AG052821 (BP); and the Foundation for Anesthesia Education and Research: FAER MRTG-CT-02/15/2010 (BP). ShiNung Ching holds a Career Award at the Scientific Interface from the Burroughs-Wellcome Fund.

## TECHNICAL TERMS

General anesthesia: Pharmacologically induced state of unconsciousness or unresponsiveness.
Brain network dynamics: Time-varying changes in connectivity and patterns of activation among distinct brain regions.
Wakefulness: Behavioral regime in which a person actively responds to external stimulation of the senses.
Maximum entropy principle: A model for the activity of a system should assume as little as possible beyond prior information and measured data.
Ising model: Network model in which nodes take binary states and energy is computed based on pairwise interactions, originally applied to ferromagnetism.
Free-energy principle: Postulates that as a general rule, the brain tends to move toward states having lower energy.
Functional connectivity: Mathematical description of the relationship between brain regions, typically in terms of correlations.
Resting-state networks: Subnetworks in the brain consisting of regions of shared functional specialization when the subject is not engaged in an active task.
Sevoflurane: Sedative drug used to administer general anesthesia by inhalation.
BOLD fMRI: Neuroimaging method in which brain activity is estimated by measuring variations in blood oxygen content via nuclear magnetic resonance.
