# Supplementary material for: High-energy brain dynamics during anesthesia-induced unconsciousness
Source: Netw Neurosci. 2017 Dec 1;1(4):431–45. doi: 10.1162/NETN_a_00023 (PMC6063715; doi:10.1162/NETN_a_00023)
Supplement: Supplementary file 1 [file netn-01-431-s001.pdf]

Riehl, J. R., Palanca, B. J., & Ching S. (2017). Supporting Information for “High-energy brain dynamics during anesthesia-induced unconsciousness.” Network Neuroscience, 1(4), 431-445. https://doi.org/10.1162/netn_a_00023

High-energy brain dynamics during anesthesia-induced unconsciousness: Supplementary materials

ROBUSTNESS TO CONNECTION DENSITY

In the main results, we included all edges in the functional connectivity networks, meaing that even very weakly correlated regions can contribute small amounts to the overall energy. To quantify the robustness of our observations to the deletion of weakly correlated edges, we computed the energy distributions after thresholding the functional connectivity network such that 0.7, 0.5, and 0.3 of the total number of edges are included in the network. In this supplementary section, we provide the energy distributions associated with Fig. 5 in the main document.

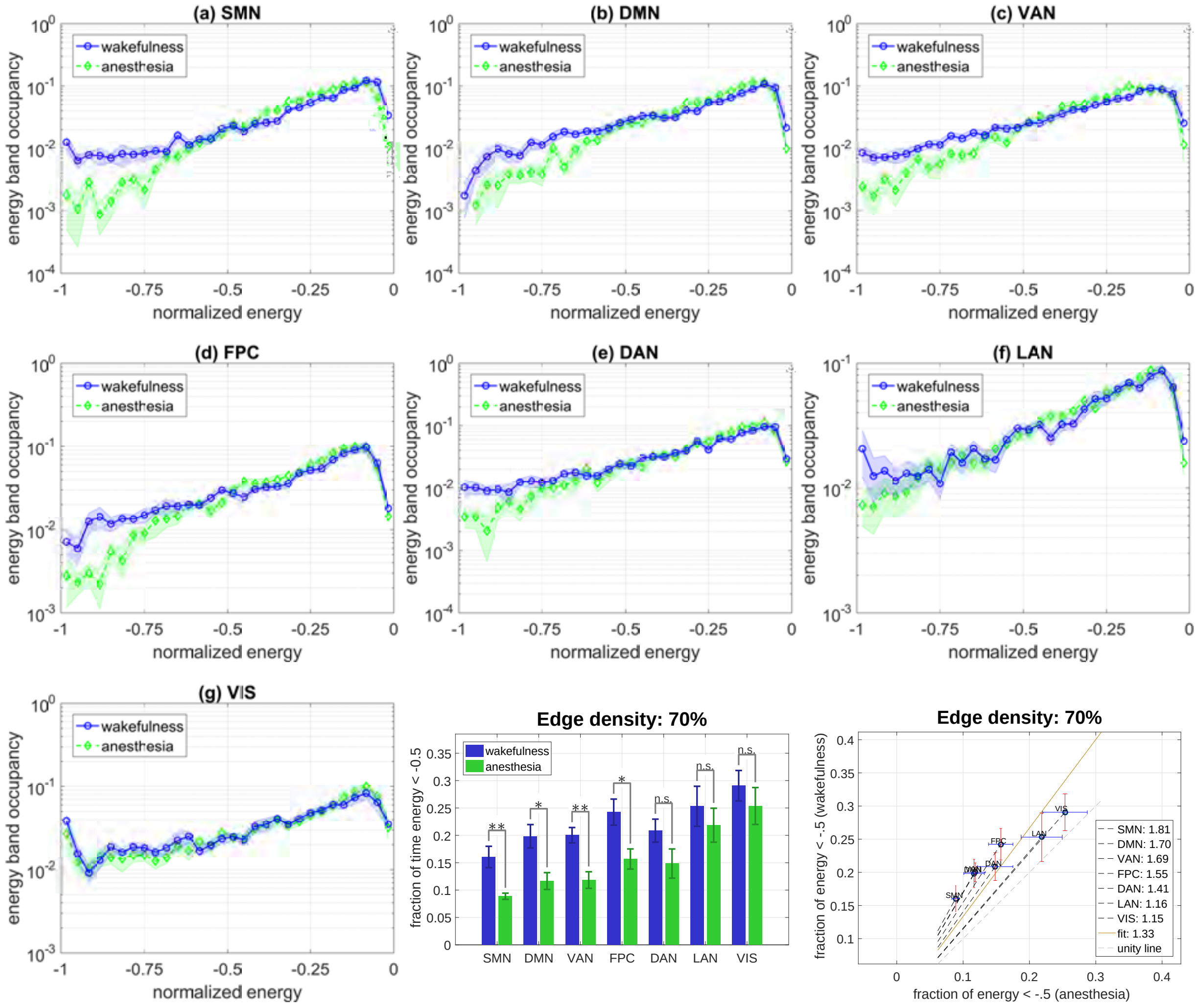

Figure 1. Same analysis as Fig. 3 in main text, but with functional connectivity network thresholded such that 70% of all possible edges are included.

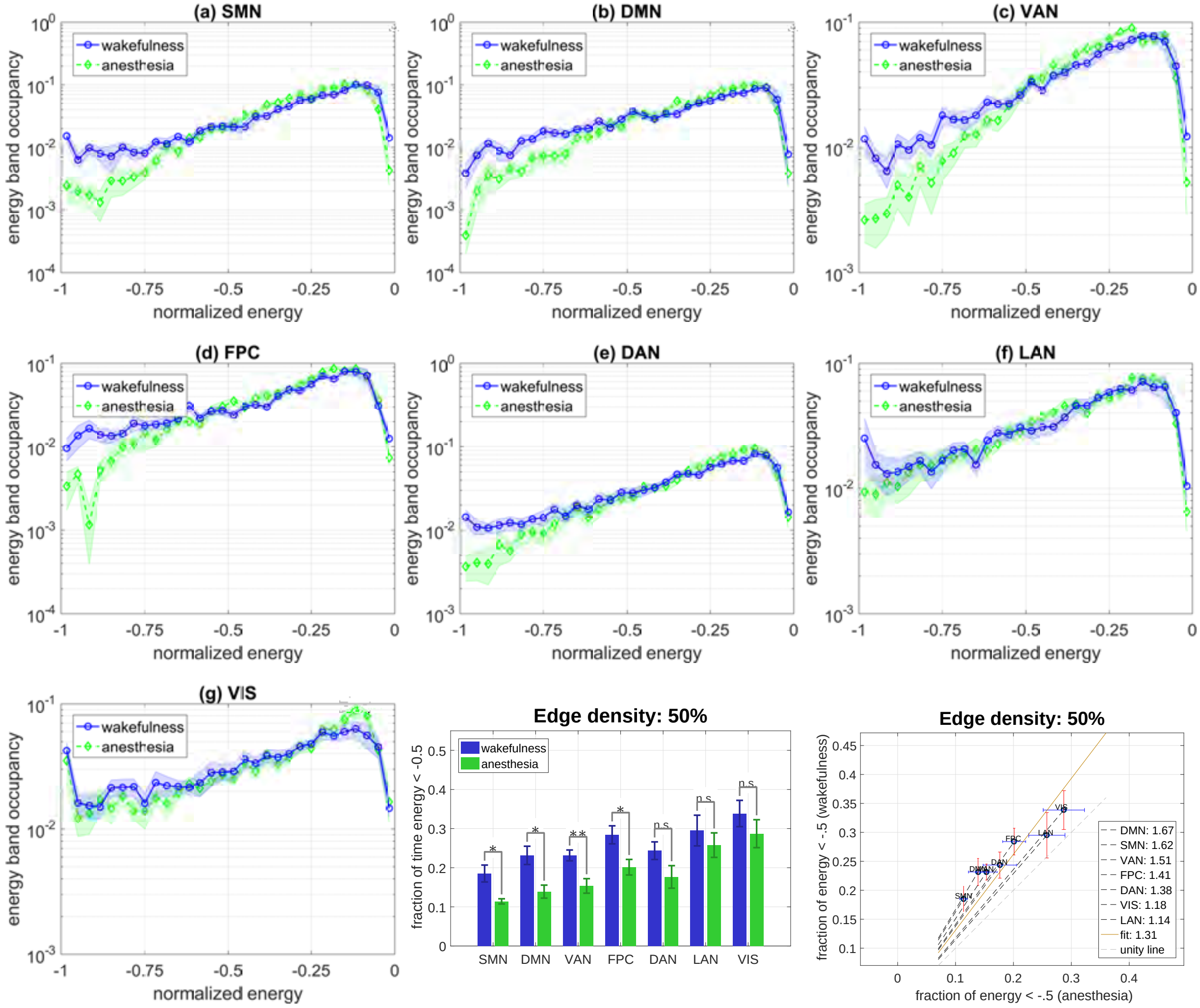

Figure 2. Same analysis as Fig. 3 in main text, but with functional connectivity network thresholded such that 50% of all possible edges are included.

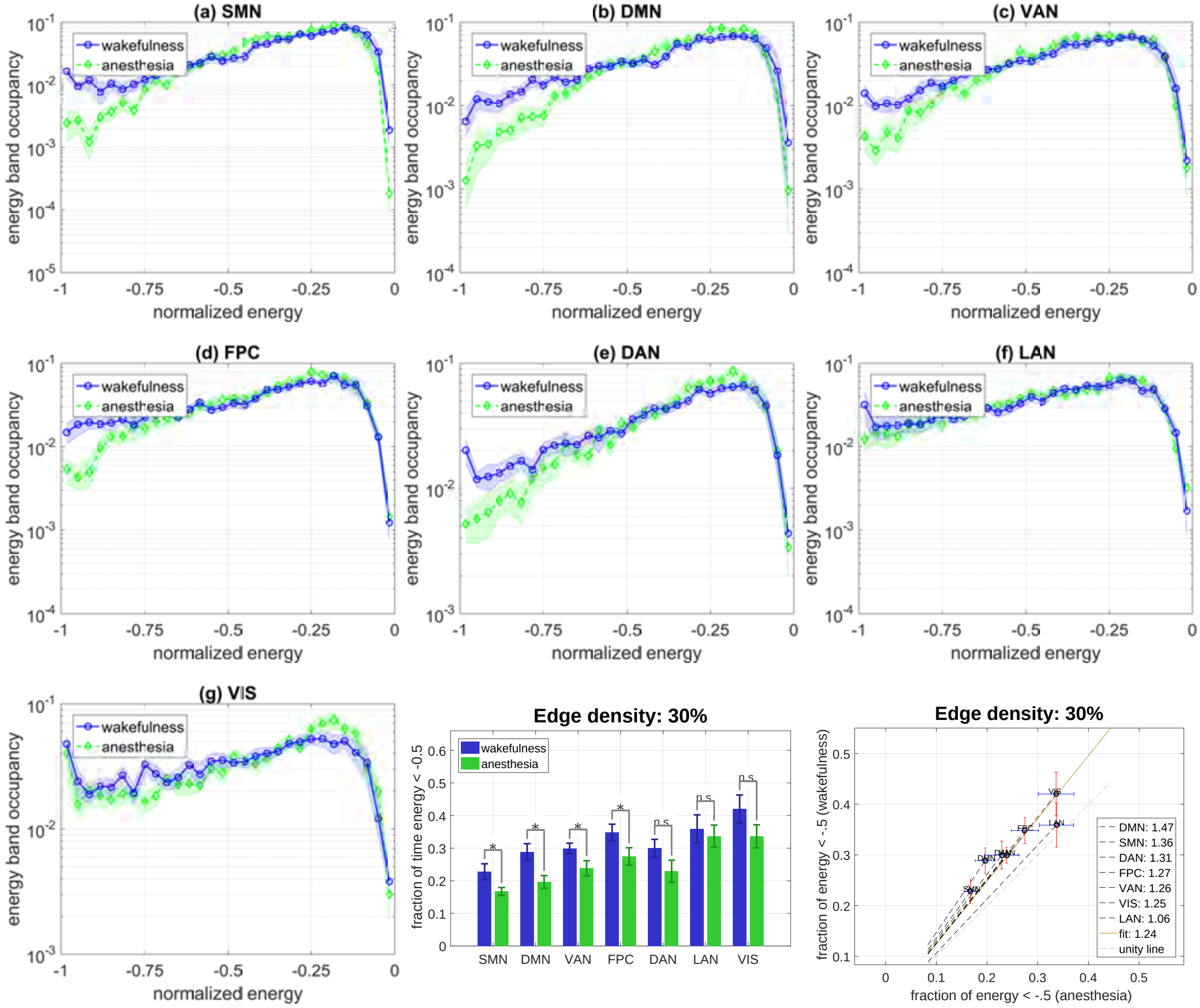

Figure 3. Same analysis as Fig. 3 in main text, but with functional connectivity network thresholded such that 30% of all possible edges are included.

ENERGY NORMALIZATION

The primary reason for using the normalized Ising Hamiltonian rather than the standard unnormalized one was to remove a potential source of bias. Since the correlations are lower across all RSNs during anesthesia, this would most likely have a direct effect on the energies, if not removed via normalization. Nevertheless, it may still be informative to see the difference in raw energies between the two conditions. We show the results in Fig. 5. Indeed, there is an even greater difference in energies between the two conditions for most RSNs, with the exception of VIS and LAN.

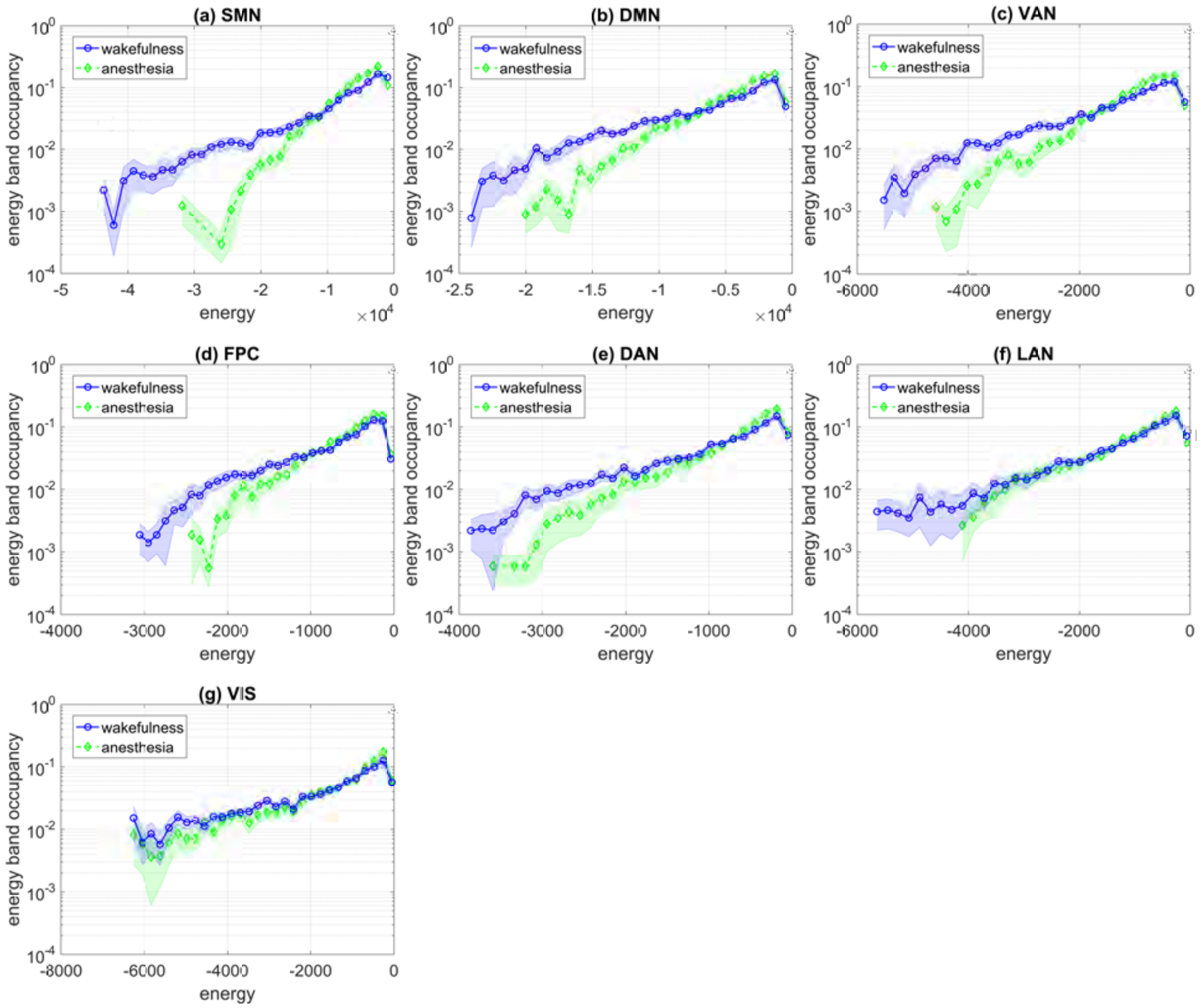

4 **Figure 4.** Same analysis as Fig. 3 in main text, but using the standard unnormalized Ising energy.

VOLUNTEERS WITH PAIRED DATA

In the original analysis, we chose to include all usable data, which meant including only wakefulness data from three volunteers and only anesthetized for three others. Here, we discard that data and examine data from only those volunteers where data from both conditions is available. For this case, we can make the same general observations, with an even greater contrast appearing in several of the RSNs.

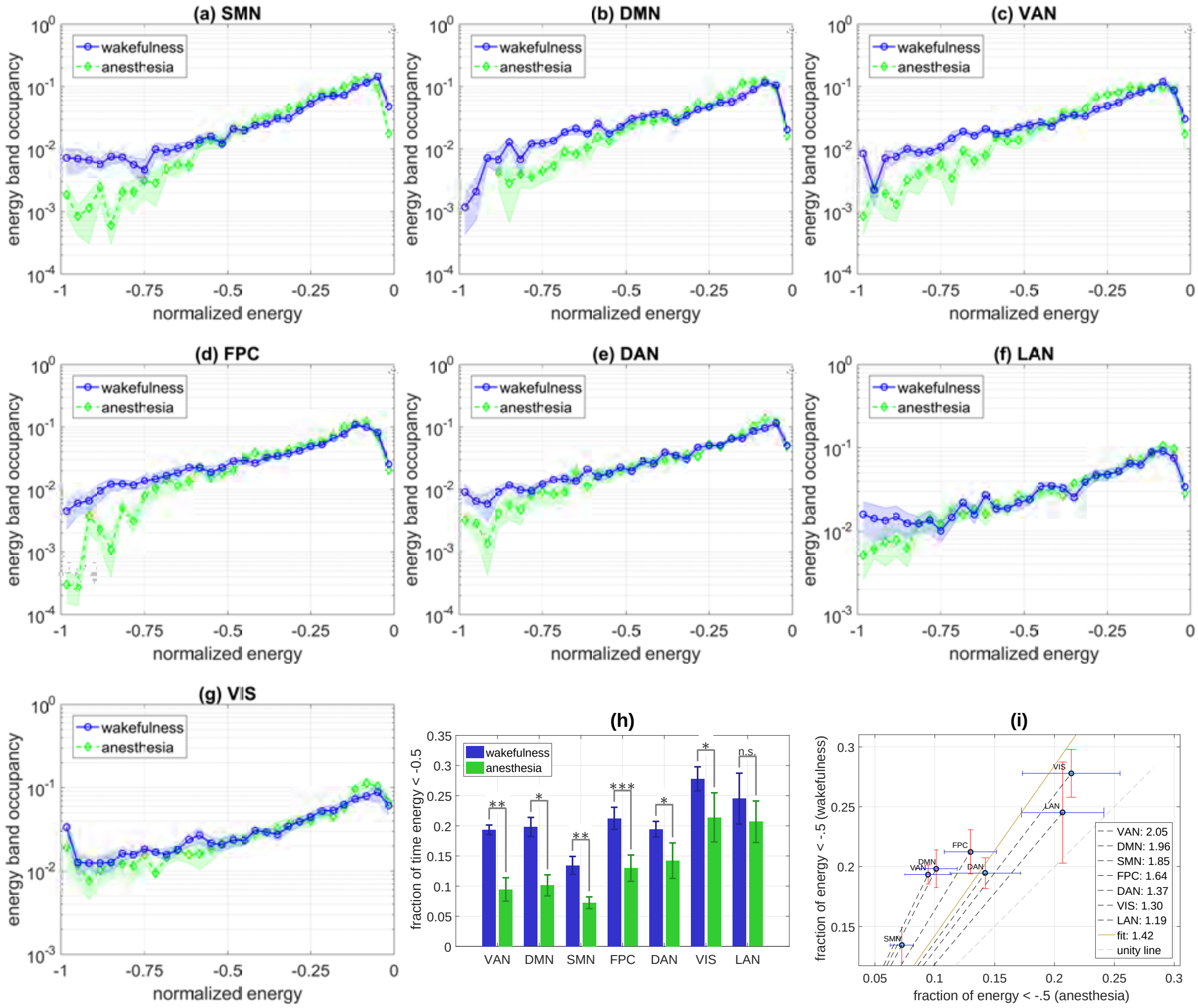

5 **Figure 5.** Same analysis as Fig. 3 in main text, but using the standard unnormalized Ising energy.
